# Human Cardiosphere-Derived Cells from Patients with Chronic Ischaemic Heart Disease Can Be Routinely Expanded from Atrial but Not Epicardial Ventricular Biopsies

**DOI:** 10.1007/s12265-012-9389-0

**Published:** 2012-07-03

**Authors:** Helen H. L. Chan, Zaal Meher Homji, Renata S. M. Gomes, Dominic Sweeney, George N. Thomas, Jun Jie Tan, Huajun Zhang, Filippo Perbellini, Daniel J. Stuckey, Suzanne M. Watt, David Taggart, Kieran Clarke, Enca Martin-Rendon, Carolyn A. Carr

**Affiliations:** 1 Department of Physiology, Anatomy and Genetics, University of Oxford, Sherrington Building, Parks Road, Oxford, OX1 3PT UK; 2 Stem Cell Research Laboratory, NHS Blood and Transplant, John Radcliffe Hospital, Oxford, UK; 3 Nuffield Department of Clinical Laboratory Sciences, University of Oxford, John Radcliffe Hospital, Oxford, UK; 4 Nuffield Department of Surgical Sciences, University of Oxford, John Radcliffe Hospital, Oxford, UK; 5 Present Address: Advanced Medical and Dental Institute, Universiti Sains Malaysia, Penang, Malaysia

**Keywords:** Cardiosphere-derived cells, Ischaemic heart disease, Diabetes, Human

## Abstract

To investigate the effects of age and disease on endogenous cardiac progenitor cells, we obtained right atrial and left ventricular epicardial biopsies from patients (*n* = 22) with chronic ischaemic heart disease and measured doubling time and surface marker expression in explant- and cardiosphere-derived cells (EDCs, CDCs). EDCs could be expanded from all atrial biopsy samples, but sufficient cells for cardiosphere culture were obtained from only 8 of 22 ventricular biopsies. EDCs from both atrium and ventricle contained a higher proportion of c-kit+ cells than CDCs, which contained few such cells. There was wide variation in expression of CD90 (atrial CDCs 5–92 % CD90+; ventricular CDCs 11–89 % CD90+), with atrial CDCs cultured from diabetic patients (*n* = 4) containing 1.6-fold more CD90+ cells than those from non-diabetic patients (*n* = 18). No effect of age or other co-morbidities was detected. Thus, CDCs from atrial biopsies may vary in their therapeutic potential.

**Electronic supplementary material:**

The online version of this article (doi:10.1007/s12265-012-9389-0) contains supplementary material, which is available to authorized users.

## Introduction

Cardiovascular disease remains the leading cause of death in the Western world [1]. Cardiac stem/progenitor cells, identified in the heart in 2003 [2, 3], are primed to repair damaged myocardium. To provide sufficient cells for therapy, cardiac stem/progenitor cells can be expanded in vitro, by selection using cell surface markers such as c-kit [2] or sca-1 [3], or from explanted biopsies via the formation of cardiospheres [4, 5]. Stem/progenitor cells isolated using each of these methods have improved cardiac function in animal models [2, 3, 5, 6].

Cardiosphere-derived cells (CDCs) are an heterogeneous population, comprising c-kit+/CD105+ cells, CD90+/CD105+ cells and a small number of CD31+ and CD34+ progenitor cells [7]. In 2009, Andersen et al. suggested that CDCs did not contain cardiac stem cells but were a combination of cardiac fibroblasts and CD45+ blood-borne cells [8]. However, this was rebutted by Davis et al. [7], who demonstrated that c-kit+, CD31+/CD34+ and CD90+ explant-derived cells (EDCs) could be cultured from human, mouse, rat and pig hearts and that rat CDCs were clonogenic and exhibited multilineage potential. Furthermore, they showed that human CDCs, when expanded from endomyocardial biopsies and transplanted into the infarcted mouse heart, differentiated into cardiomyocytes, endothelial and smooth muscle cells [9]. However, it is still uncertain whether biopsy location, increasing grades of cardiac failure or the presence of co-morbid risk factors, such as diabetes or hypertension, can affect the number and characteristics of the CDC population. Markers of cell senescence increase with increasing age and with type 1 diabetes in c-kit+ cells from human and mouse hearts [10–12], suggesting that this population of the cardiac stem/progenitor cells may be susceptible to damage.

CDCs have recently been tested in a phase 1 clinical trial, in patients 2–4 weeks after acute myocardial infarction (CADUCEUS trial) [13]. Selected c-kit+ progenitor cells have also been tested in patients with ischaemic cardiomyopathy (SCIPIO trial) [14]. As the heart contains few c-kit+ cells (approximately 1 in 10,000 myocytes [2]), it took 3 to 4 months to culture 1 million c-kit+ cells for the SCIPIO trial [14]. The cells were tested to confirm high expression of c-kit and low indicators of senescence. In contrast, for the CADUCEUS trial, 25 million CDCs were cultured in 36 days. The CDCs were assessed by flow cytometry to confirm high expression of CD105 and low numbers of CD45+ cells. The proportion of c-kit+ cells was not reported. Despite the higher number of cells administered in the CADUCEUS trial, and a significantly reduced infarct size at 6 months, there was no significant improvement in left ventricular ejection fraction (LVEF). Conversely, the SCIPIO trial showed an improvement in LVEF in patients after 4 months, suggesting that selected c-kit+ cells may be more effective than the heterogeneous CDC population.

Here, we cultured CDCs from the atria and ventricles of patients undergoing cardiac bypass surgery to characterise the cell population. We also assessed the effect of increasing severity of cardiac failure, and the existence of co-morbidities, such as diabetes and hypertension, on the number and characteristics of the CDC population obtained.

## Materials and Methods

### Biopsy Collection

Full-thickness right atrial biopsies and left ventricular epicardial biopsies were obtained at the John Radcliffe Hospital, Oxford, from coronary artery bypass graft patients, with informed written consent. Ethical approval was granted by the relevant Research Ethics Committee to obtain cardiac biopsies and conduct this study (REC reference: 07/H0607/95), which was carried out in accordance with the Helsinki Declaration of 1975, as revised in 2000. All human tissue samples and cells were handled, processed and stored under a Human Tissue Authority licence.

### Culture of CDCS

Biopsies were placed in Complete Explant Medium (CEM, see Electronic Supplementary Material) on ice and processed within 2–3 h. Gross connective and adipose tissues were removed to leave only cardiac tissue. After washing twice with phosphate buffered saline (PBS, Invitrogen, UK), the sample was cut into 5-mm segments and digested in 0.05 % trypsin (Invitrogen) for 3 min at room temperature. The segments were further minced into 1-mm fragments that were washed again in PBS and plated out as explants onto fibronectin-coated (Sigma, USA) 60-mm Petri dishes (Corning, UK) containing 0.5 mL of CEM. Explants were incubated for 1 h at room temperature to allow adhesion of explants to the fibronectin coating, before a further 1 mL of CEM was added. Explants were cultured at 37 °C in 5 % CO_2_, with the CEM replaced every 4 days. A layer of long thin fibroblast-like cells spontaneously emerged from edges of adherent explants, followed by overlying round phase bright cells. Phase bright cells were harvested once confluent by washing explants with PBS, with 1 mL 0.53 mM EDTA (Versene, Invitrogen), then treating enzymatically with 1 mL trypsin for 5–7 min at 37 °C. An additional wash of PBS ensured complete removal of phase bright cells in addition to some fibroblasts. Explants could be harvested twice, allowing 1 week between harvests. Harvested cells were seeded into poly-d-lysine-coated wells at a concentration of 3 × 10^5^ cells in 500 μL of Cardiosphere Growth Medium (see Electronic Supplementary Material). Fully formed, loosely adherent cardiospheres were harvested by gentle pipetting and plated onto fibronectin-coated T75 flasks (Corning) for expansion as CDCs to passage 2.

### Flow Cytometry

Once confluent at passage 2, CDCs were harvested using trypsin (5 min at 37 °C) after washing three times with PBS and once with Versene. Non-specific binding of antibodies was blocked using human FcR block (Miltenyi Biotec., Germany) at a concentration of 100 μL/1 × 10^6^, incubated on ice in the dark for 30 min. Cells were washed, suspended in PBS to a final concentration of 2 × 10^6^ cells/mL and incubated with the appropriate antibody (see Electronic Supplementary Material) for flow cytometric analysis using a BD LSRII flow cytometer (BD Biosciences, UK) equipped with UV, blue and red lasers.

### Cardiomyogenic Differentiation

Cardiomyogenic differentiation was induced using cardiomyocyte differentiation medium (CDM) (2 % FBS ESQ (embryonic stem cell-qualified; Invitrogen), 1 % insulin transferrin selenium in IMDM:DMEM/F12 (1:1, Sigma) supplemented with 1 mM dimethyl sulphoxide (DMSO). The DMSO-supplemented CDM was changed every 2 days for 6 days. Then, all cells were aspirated with PBS to remove the dead cells, and 2 ml CDM supplemented with 0.1 mM ascorbic acid was added to the plate. The medium was changed every 2 days for the following 6 days.

### Immunocytochemistry

Conditioned CDCs were grown on Nunc Lab-Tek® 4-well chamber slides precoated with 10 μg/ml fibronectin and fixed with 4 % paraformaldehyde (Sigma) for 10 min on ice. Fixed cells were blocked with 10 % donkey serum (Biosera, UK) in 0.1 % PBS-tween for an hour at room temperature and then incubated with the primary antibody (see Electronic Supplementary Material) diluted in PBS, overnight at 4 °C in a humidified chamber. Cells were incubated with the appropriate secondary antibody for an hour at 4 °C and the immunofluorescence detected using a confocal microscope (Zeiss Confocal LSM 520 META).

For filamentous actin staining, a rhodamine phalloidin probe was used. Cells were permeabilised with 0.1 % Triton after fixation and blocking for 30 min using a solution of 2 % FBS + 2 % BSA in PBS. Rhodamine phalloidin (Invitrogen) was diluted in PBS and added to the cells for 15 min before washing and mounting.

### Statistical Analysis

Data are expressed as means ± standard error. Analysis of variance, with a Tukey post hoc test, *T* tests and Pearson correlations were performed using Excel and SPSS software. Statistical significance was assumed to be *p* < 0.05.

## Results

### Patient Demographics

Full-thickness right atrial biopsies (*n* = 22; 468 ± 40 mg) and left ventricular epicardial biopsies (*n* = 22; 164 ± 29 mg) were taken from patients undergoing coronary artery bypass surgery (40–83 years of age), three of whom underwent concurrent valve replacement. Details of the patient population are given in Table 1.

**Table 1:** Patients’ demographics

Age	67 ± 2
BMI	28 ± 1
Sex (male)	20 (91 %)
Class I	1 (0.05 %)
Class II	11 (50 %)
Class III	7 (32 %)
Class IV	2 (9 %)
Missing	1 (4.5 %)
Diabetes	4 (18 %)
Smoker	9 (41 %)
Hypertension	15 (68 %)
High cholesterol	19 (86 %)
Aspirin	15 (68)%
ACE inhibitors	12 (55)%
Beta blockers	15 (68 %)
Statins	20 (90 %)

Data are *n* (percent) or mean (SEM)

*BMI* body mass index, *NYHA* New York Heart Association

### Cell Culture

Biopsies were explanted onto fibronectin-coated dishes for culture of explant-derived cells (EDCs). A bed of fibroblast-like cells grew out from the explants, over which phase-bright cells migrated. Once confluent, EDCs could be harvested for culture as cardiospheres.

**Fig. 1:**
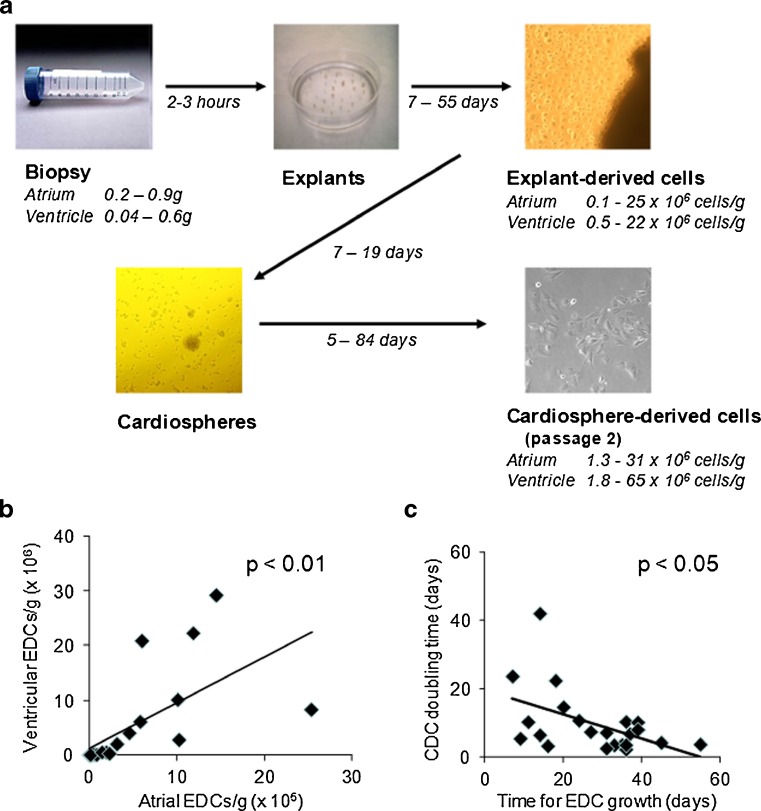
Expansion of EDCs and CDCs from atrial and ventricular biopsies. **a** Considerable variation was observed in the time taken for culture of confluent explant- and cardiosphere-derived cells and in the number of cells obtained. **b** The number of EDCs generated from atrial biopsies correlated with the number from ventricular biopsies. **c** The time taken for atrial EDCs to reach confluence correlated inversely with the doubling time of CDCs

The time taken for each stage of the cell culture process and the number of cells produced varied considerably (Fig. 1a, Table S1). Cardiospheres grow slowly when EDCs are seeded at a low density [15], so at least 40,000 EDCs need to be harvested for successful cardiosphere culture. All atrial biopsies generated sufficient EDCs for cardiosphere formation, over 7 to 55 days, but it was only possible to culture cardiospheres from eight ventricular biopsies (denoted group AV).

There was a significant correlation between the number of EDCs produced from atrial and ventricular biopsies from the same patients (Fig. 1b). The time taken to culture confluent atrial EDCs inversely correlated with the doubling time of the resultant CDCs, in that fast growing EDCs generated fast-growing CDCs (Fig. 1c). The low sample number prevented confirmation of a similar result for ventricular EDCs.

There were no correlations between the rate of growth or the number of EDCs or CDCs with age or disease (Table 2).

**Table 2:** Number of EDCs and CDCs produced by atrial biopsies, time taken for growth, and cell surface markers divided by age or disease

	All	Age < 65	Age > 65	Non-diabetic	Diabetic	Normo-tensive	Hyper-tensive	Normal cholesterol	High cholesterol	NYHA 1 or 2	NYHA 3 or 4
	*n* = 22	*n* = 10	*n* = 12	*n* = 18	*n* = 4	*n* = 6	*n* = 16	*n* = 3	*n* = 19	*n* = 12	*n* = 10
Time for EDC growth (days)	28 ± 3	26 ± 3	29 ± 5	27 ± 3	30 ± 6	35 ± 7	25 ± 3	35 ± 1	27 ± 3	25 ± 4	32 ± 5
EDC/g (×10^6^)	4.7 ± 1.4	3.8 ± 1.4	5.4 ± 2.4	5.3 ± 1.7	1.9 ± 1.1	5.3 ± 4.4	4.4 ± 1.2	10.8 ± 3.1	3.7 ± 1.4	3.7 ± 1.6	5.9 ± 5
CDC/g (×10^6^)	8.9 ± 1.7	9.2 ± 3.8	8.6 ± 1.3	8.1 ± 1.7	12.3 ± 6.8	6.5 ± 2.7	9.7 ± 2.2	8.8 ± 4.2	8.9 ± 2.0	7.6 ± 2.3	10.3 ± 2.9
Doubling time (days)	10 ± 2	9 ± 2	10 ± 3	10 ± 3	6 ± 1	5 ± 1	11 ± 3	3 ± 1	11 ± 2	12 ± 4	6 ± 1
CD117 (%)	0.2 ± 0.1	0.3 ± 0.2	0.1 ± 0.1	0.2 ± 0.1	0.1 ± 0.1	0.3 ± 0.2	0.2 ± 0.1	0 ± 0	0.2 ± 0.1	0.3 ± 0.2	0.1 ± 0.1
CD31 (%)	2.1 ± 0.7	2.9 ± 1.9	1.5 ± 0.4	2.3 ± 0.8	1.2 ± 0.8	1.9 ± 0.5	2.1 ± 1.0	2.9 ± 1.4	1.9 ± 0.8	2.6 ± 1.5	1.5 ± 0.5
CD34 (%)	1.2 ± 0.6	1.9 ± 1.6	0.6 ± 2	1.2 ± 0.8	1.0 ± 0.3	0.6 ± 0.42	1.4 ± 0.9	0.7 ± 0.3	1.3 ± 0.7	1.3 ± 1.1	1.0 ± 0.3
CD90 (%)	55 ± 5	53 ± 7	57 ± 8	50 ± 5	79 ± 8*	47 ± 11	58 ± 6	38 ± 10	58 ± 6	58 ± 4	52 ± 10
CD105 (%)	80 ± 4	84 ± 4	76 ± 7	78 ± 5	90 ± 8	79 ± 9	80 ± 5	94 ± 2	78 ± 5	78 ± 6	82 ± 6

*NYHA* New York Heart Association

*p < 0.05 compared with non-diabetic

### EDC and CDC Characterisation

**Fig. 2:**
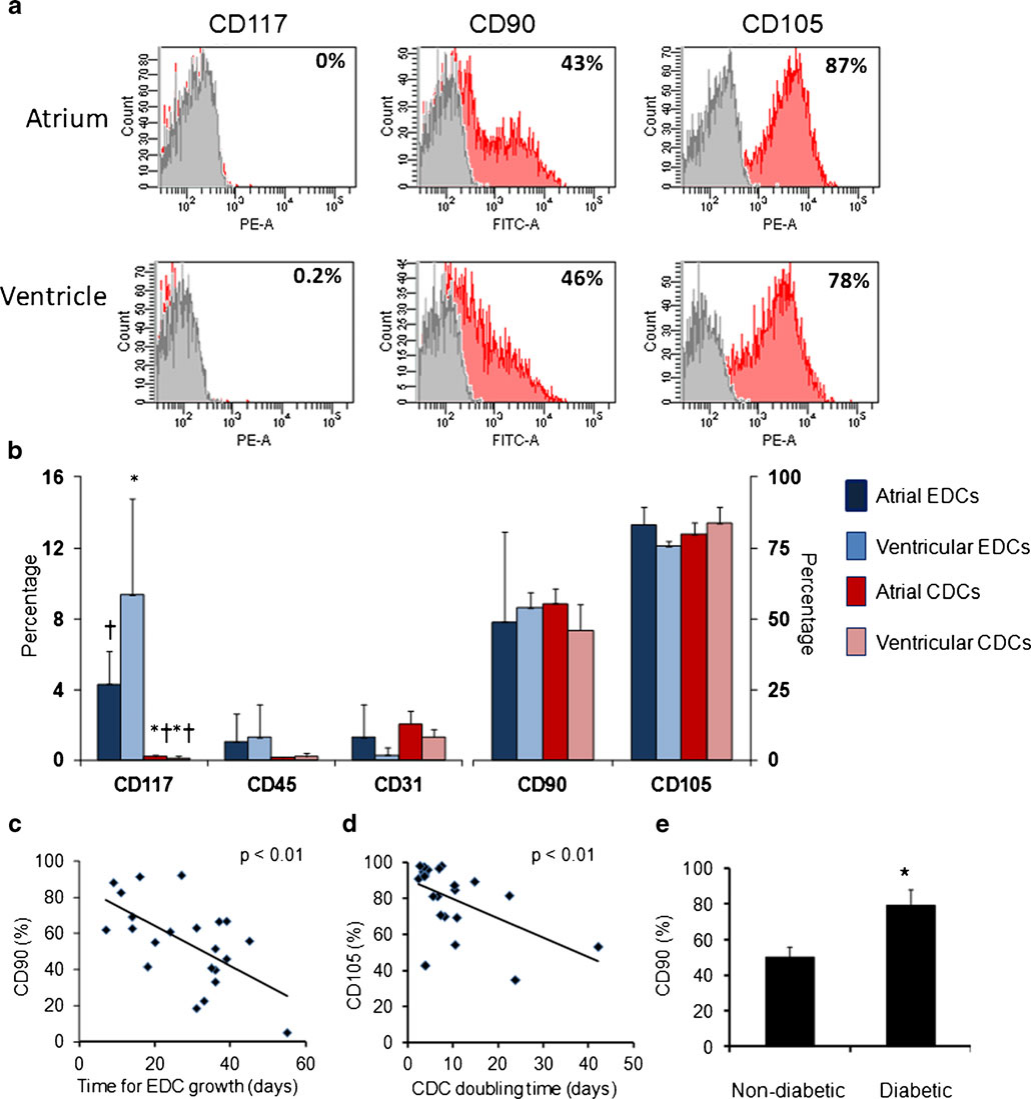
Cell surface markers on EDCs and CDCs. **a** Representative flow cytometry plots for CD117 (c-kit), CD90 and CD105 (with isotype controls in *grey*) in CDCs from atrial (*top*) and ventricular (*bottom*) biopsy samples. **b** Expression of cell surface markers by EDCs and CDCs from atrial and ventricular biopsies (*n* = 3 for atrial and ventricular EDCs, *n* = 22 for atrial CDCs and 8 for ventricular CDCs; *p < 0.05 compared with atrial EDCs, †*p* < 0.05 compared with ventricular EDCs). **c** The time taken for culture of confluent EDCs inversely correlated with CD90 expression in CDCs and **d** the doubling time of CDCs inversely correlated with CD105 expression in CDCs. **e** Atrial CDCs from diabetic patients (*n* = 4) contained significantly more CD90+ cells than those from non-diabetic patients (*n* = 18; *p < 0.05 compared with non-diabetic patients). *Error bars* show standard errors

Cell surface markers on all CDCs (*n* = 22) and a subset of EDCs (*n* = 3) were characterised using flow cytometry (Fig. 2a, b; Tables 2 and 3). EDCs and CDCs comprised predominantly of CD105+ cells, with a wide variation in expression of CD90 (atrial EDCs 26–71 %, ventricular EDCs 38–70 %; atrial CDCs 5–92 % CD90+; ventricular CDCs 11–89 % CD90, Table S1) and with low expression of c-kit, CD31 and CD34. There were significantly more c-kit+ cells in EDCs than CDCs, from both atrial and ventricular biopsies, and ventricular EDCs contained more c-kit+ cells than atrial EDCs (Fig. 2b; Table 3). EDCs contained 1 % CD45+ cells, which were not detected in the CDC population. There were no other significant differences in expression of cell surface markers in EDCs or CDCs from atrial tissue compared with those from ventricular tissue (Fig. 2b).

**Table 3:** Cell surface markers on EDCs and CDCs from atrial and ventricular biopsies, analysed using flow cytometry

Marker	EDCs		CDCs		CDCs	
	Atrial (*n* = 3)	Ventricular (*n* = 3)	Atrial (*n* = 22)	Ventricular (*n* = 8)	Atrial group AV (*n* = 8)	Atrial group A (*n* = 14)
CD117 (c-kit)	4 ± 2 %*	9 ± 5 %**	0.2 ± 0.1 %* **	0.1 ± 0.1 %* **	0 %* **	0.3 ± 0.1 %* **
CD45	1.1 ± 1.4 %	1.3 ± 1.8 %	0. %	0 %	0 %	0 %**
CD31	2.6 ± 1.8 %	0.6 ± 0.4 %	2.1 ± 0.7 %	1.3 ± 0.4 %	1.7 ± 0.7 %	2.3 ± 1.3 %
CD34			1.2 ± 0.6 %	0.6 ± 0.5 %	0.9 ± 0.3 %	1.4 ± 1.0 %
CD133			0 %	0 %	0 %	0 %
CD90	49 ± 32 %	54 ± 23 %	55 ± 5 %	46 ± 9 %	42 ± 9 %	63 ± 5 %***
CD105	83 ± 6 %	76 ± 1 %	80 ± 4 %	84 ± 6 %	83 ± 7 %	78 ± 5 %

Group AV indicates hearts from which ventricular CDCs were cultured, group A indicates hearts from which no ventricular CDCs could be obtained

*p < 0.05 compared with ventricular EDCs; **p < 0.05 compared with atrial EDCs; ***p < 0.05 compared with group AV

The percentage of CD90+ CDCs inversely correlated with the time taken to culture confluent EDCs, indicating that where biopsies produced confluent EDCs relatively rapidly, these EDCs contained more CD90+ cells (Fig. 2c). Predominantly, the atrial biopsies with rapid outgrowth came from hearts from which insufficient ventricular EDCs were produced (denoted group A). CDCs from group A contained 21 % more CD90+ cells than those from group AV (Table 3). Furthermore, the percentage of CD105+ CDCs inversely correlated with the CDC doubling time (Fig. 2d), suggesting that the doubling time of CD105+ cells is faster than that of CD105− cells.

Atrial CDCs from diabetic patients (*n* = 4) contained significantly more CD90+ cells (79 ± 8 %) than those from non-diabetic patients (50 ± 5 %; *n* = 18; Fig. 2e), but there was no other correlation between age or disease and CDC numbers, doubling time or cell surface markers (Table 2).

### CDC Differentiation

**Fig. 3:**
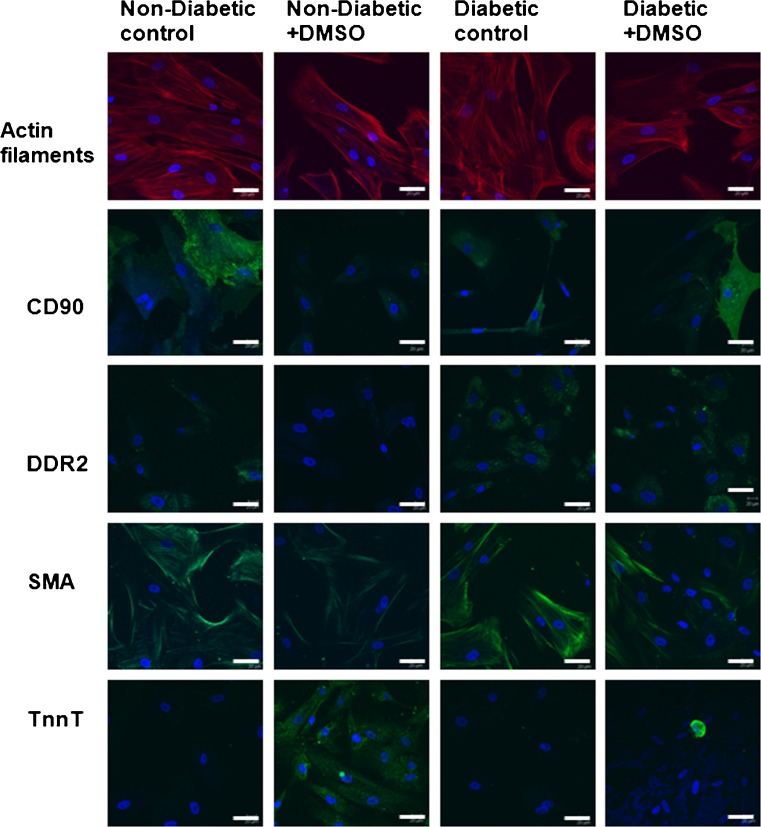
Immuno-staining of CDCs. Representative confocal images from immuno-staining of atrial CDCs for actin filaments, *CD90*, discoidin domain receptor 2 (*DDR2*), smooth muscle actin (*SMA*) and troponin-T (*TnnT*). Cells from diabetic patients (*right 2 panels*) contained higher levels of the fibroblast marker DDR2 than those from non-diabetic patients (*left 2 panels*) and showed low expression of TnnT after treatment with differentiation medium containing dimethyl sulphoxide (*DMSO*) for 2 weeks. Scale bars = 50 μm

To further investigate differences between CDCs from diabetic and non-diabetic patients, we treated CDCs from non-diabetic (*n* = 2) or diabetic patients (*n* = 2) with cardiomyogenic differentiation medium for 2 weeks. Untreated and treated CDCs were stained for CD90, the fibroblast marker discoidin domain receptor 2 (DDR2), smooth muscle actin (SMA) and troponin T (TnnT) (Fig. 3). Confirming the flow cytometric analysis, untreated CDCs from diabetic patients contained more CD90+ cells than those from non-diabetic patients and also contained more cells positive for DDR2. Untreated CDCs also contained cells expressing smooth muscle actin (SMA) but few cells positive for TnnT. Following treatment with cardiomyogenic differentiation medium, there was a decrease in the proportion of cells expressing CD90 and SMA and an increase in the number of cells positive for TnnT, but possibly to a lesser extent in the CDCs from diabetic patients.

## Discussion

Here, we show that CDCs cannot be cultured routinely from ventricular epicardial biopsies from patients with ischaemic heart disease. Furthermore, both atrial and ventricular epicardial CDCs contain variable numbers of CD90+ cells and few c-kit+ cells.

As has been seen with studies in bone marrow stem cells, there is a disparity between results observed in the single human clinical trial using CDCs [13] and those in animal models [4–6, 16] where cells were predominantly isolated from young, healthy animals rather than those with heart disease. Additionally, in animal models, there are no potentially confounding pharmacological or surgical treatments, whereas in the clinic, ethical practice mandates that cells be administered in addition to current ‘best practice’ therapy. Here, we found that CDCs from patients with ischaemic heart disease contained few c-kit+ cells and an increased proportion of CD90+ cells than reported originally [5] (Fig. 2). There are now at least 12 papers reporting cell surface markers on cells expanded from human biopsies using the cardiosphere protocol (Table 4), although not all give details of patient age or disease. While there is consensus that the majority of cells are CD105+, the proportions of c-kit+ and CD90+ cells vary considerably. The effect of co-morbid risk factors such as diabetes and hypertension on the types and proportions of cells obtained has not been reported, although it is likely that at least some of the variation results from differences in culture protocols between laboratories, as small changes to the length of time before harvest of EDCs and of culture of cardiospheres may affect the resultant CDC population, even within laboratories [17, 18].

**Table 4:** Cell surface markers reported for cells cultured from human biopsy samples using the cardiosphere protocol

First author	Year	Cell type	Patient age	Patient number	Biopsy location	c-Kit	CD90	CD105	CD31	CD34	Troponin	Vimentin/collagen 1
Davis [7]	2009	EDC	32 ± 12 years	59	Endomyocardium	∼12 %	∼32 %			∼5 %		
			49 ± 15 years	12								
		Csp				pos	pos	pos	pos	neg	pos	pos for vimentin
Zakharova [29]	2010	EDC	53–73 years	Not given	Atrium	∼31 %					∼34 %	∼28 % vimentin
		Csp				32 %					28 %	6 % vimentin
Messina [4]	2004	Csp	1 month–80 years	8	Atrium/ventrical	pos			pos	pos	pos	
Maxeiner [36]	2010	EDC/Csp	0.2–9 years	6	Not given	mRNA but no protein						neg for vimentin
			24–81 years	4								
Li [37]	2010	Csp		11	Endomyocardium	17 %						
		CDC				5 %	18 %	100 %				
Smith [5]	2007	CDC	31 ± 2 years	59	Endomyocardium	∼15 %	∼40 %	∼95 %	∼4 %	∼10 %		
			47 ± 4 years	11								
Tateishi [22]	2007	CDC	9 days–77 years	18	Not given	0.8 %	68 %	99 %	0.4 %	0.5 %		Csp pos for vimentin and collagen 1
Barth [17]	2008	CDC		28	Endomyocardium	13 %	34 %	99 %	9 %	6 %		
Konincx [24]	2010	CDC	68 ± 7 years	12	RAA	neg	Partial expression					
Mishra [28]	2011	CDC	Neonate	118	RAA	9 %	55 %	85 %				neg for collagen I
			Infant			7 %	70 %	80 %				
			2–13 years			3 %	70 %	80 %				
Li [18]	2012	CDC			Endomyocardium	7 %	18 %	100 %	0.6 %	1 %	9 %	
Makkar [13]	2012	CDC	54 ± 2.5 years	31	Endomyocardium			98 %				

Approximate values are estimated from published graphs

*pos* positive, *neg* negative

It is well documented that c-kit+ cardiac stem/progenitor cells are clonogenic and able to differentiate into cells of the cardiac lineage [19–21]. Human CDCs containing few c-kit+ cells are also capable of differentiation towards the cardiomyocyte lineage, as has been shown here and by others [22–24] (Fig. 3). However, the increased proportion of CD90+ cells seen in CDCs from diabetic patients and in fast-growing CDCs may indicate that these cells contain a greater proportion of cardiac fibroblasts, as suggested by staining for DDR2 (Fig. 3). Increased fibrosis is observed in the ischaemic [25] and diabetic heart [26] and with age [27], increasing the likelihood of culturing fibroblasts within the CDC population. Here, we found no correlation between age and the proportion of CD90+ cells, but this may be because we did not isolate CDCs from younger, non-ischaemic hearts. Mishra et al. expanded CDCs from the hearts of children with congenital, non-ischaemic heart defects [28] which contained 55-70 % CD90+ cells and showed a decline in the number of c-kit+ cells with age (Table 4). They also showed that administration of CDCs to the infarcted mouse heart improved cardiac function compared with administration of cardiac fibroblasts, emphasising the need to minimise the cardiac fibroblast population in CDCs. Although the formation of cardiospheres was proposed to enhance the stem cell population of EDCs, many cell types have now been shown to form spheres, including myofibroblasts and bone marrow and dermal mesenchymal cells [24]. However, Zakharova et al. found that EDCs from atrial biopsies obtained from patients undergoing cardiac bypass surgery contained high levels of vimentin-positive cells, but that the proportion of these cells decreased after cardiosphere culture [29].

All atrial biopsies yielded CDCs, but we found that only 8 out of 22 ventricular epicardial biopsies yielded sufficient EDCs for CDC culture. Although the epicardium contains progenitor cells, epicardial biopsies require stimulation for significant outgrowth to occur [30]. It may be that the ventricular biopsies from which EDCs were cultured here contained significant amounts of myocardial tissue. Atrial CDCs from hearts from which no ventricular CDCs were cultured contained more CD90+ cells, as did CDCs from rapid-growing EDCs. Atrial tissue contains more fibroblasts than ventricular tissue, and atrial fibroblasts proliferate more rapidly than those from the ventricle [31]. More work is required to establish whether rapid-outgrowth EDCs contain more fibroblasts than those that take longer to migrate from the explant. It is thought that cardiac stem cells predominantly reside in the atria and apex of the heart [32], although we found significally more c-kit+ cells in the small subset of EDCs from the ventricle than in those from the atrium. For the CADUCEUS trial [13], 25 million CDCs were cultured within 36 ± 6 days from biopsies taken from the endomyocardial septum. Interestingly, here, no atrial or epicardial biopsy yielded that number of CDCs and the calculated time to reach 25 million CDCs ranged from 47 to 278 days (Table S1). For the SCIPIO trial [14], c-kit+ cells were isolated and expanded from atrial biopsies. The improvement in LVEF, measured in that study, indicated that c-kit+ cells may be more potent than other cells in the EDC or CDC populations. Here, we saw a decrease in the number of c-kit+ cells between the EDC and CDC stages of expansion. Expression of c-kit has been shown to vary with time in EDCs, peaking at about 21 days after plating in rat EDCs [33], and cardiosphere culture has been reported to increase the proportion of ckit+ cells [4]. Our data suggest that this may be lost again during expansion as a monolayer of CDCs.

Clearly, further careful characterisation of CDCs expanded from human patients is essential to establish transferable and reproducible cell culture techniques necessary for large multi-centre clinical trials. Lessons from the clinical trials using bone marrow cells have shown that changes to the conditions used for isolation and storage of cells can adversely affect their therapeutic potential [34, 35]. Thus, modification to the expansion protocol may be necessary to optimise the cell population produced from both diabetic and non-diabetic patients for maximum therapeutic effect.

## Electronic Supplementary Material

Below is the link to the electronic supplementary material.

ESM 1 (PDF 31 kb)
