# Supplementary material for: Human Cardiosphere-Derived Cells from Patients with Chronic Ischaemic Heart Disease Can Be Routinely Expanded from Atrial but Not Epicardial Ventricular Biopsies
Source: J Cardiovasc Transl Res. 2012 Jul 3;5(5):678–87. doi: 10.1007/s12265-012-9389-0 (PMC3447135; doi:10.1007/s12265-012-9389-0)
Supplement: Supplementary file 1 — (PDF 31 kb) [file 12265_2012_9389_MOESM1_ESM.pdf]

Human cardiosphere-derived cells from patients with chronic ischaemic heart disease can be routinely expanded from atrial but not epimyocardial ventricular biopsies

.

Helen HL Chan*, Zaal Meher Homji*, Renata SM Gomes, George N Thomas, Jun Jie Tan, Dominic Sweeney, Huajun Zhang, Filippo Perbellini, Daniel J Stuckey, Suzanne M Watt, Enca Martin-Rendon, David Taggart, Kieran Clarke, Carolyn A Carr

Address for Correspondence

Dr Carolyn Carr, Department of Physiology, Anatomy & Genetics, University of Oxford, UK**.**

Email: Carolyn.Carr@dpag.ox.ac.uk

Table S1: The time taken to culture EDCs and CDCs and the number of cells produced per patient, with the proportion of CD90+ cells and an estimation of the length of time required to culture the number of CDCs used in the CADACEUS trial.

Atrium								Ventricle						
Biopsy mass	EDCs per biopsy	CDCs per biopsy	CDC doubling time	Total culture time	CD90	Calculated time to 25 x 10^6 CDCS		Biopsy mass	EDCs per biopsy	CDCs per biopsy	CDC doubling time	Total culture time	CD90	Calculated time to 25 x 10^6 CDCS
g	x 10^6	x 10^6	days	days	%	days		g	x 10^6	x 10^6	days	days	%	days
0.3	0.19	8.8	3	42	92	47		0.17	0.02					
0.3	0.05	1.6	6	27	88	49		0.17	0.02					
0.2	4.32	3.1	2	45	33	52		0.09	0.07					
0.3	3.08	1.0	3	40	63	52		0.13	0.36	0.9	2	38	69	47
0.9	0.09	3.3	7	34	63	53		0.29	0.03					
0.6	6.17	19.0	7	52	19	55		0.07	0.71	3.4	5	48	35	62
0.6	3.62	5.4	4	48	23	56		0.08	1.67	2.6	5	51	53	65
0.4	4.88	3.8	4	53	52	63		0.07	1.56	1.3	8	62	47	97
0.3	4.36	2.4	4	50	41	63		0.04	1.17	2.6	4	51	38	65
0.5	0.44	6.0	8	50	92	65		0.11	0.03					
0.3	0.63	3.3	10	40	83	70		0.04	0.02					
0.6	1.31	2.2	4	61	56	76		0.30	0.08					
0.6	1.30	4.5	4	77	5	86		0.60	0.63	1.1	6	81	11	107
0.7	0.99	3.1	7	70	67	91		0.22	0.11	0.7	9	74	89	119
0.4	1.12	4.2	10	79	40	106		0.05	0.01					
0.7	0.08	0.9	11	56	61	108		0.40	0.01					
0.4	1.84	1.6	8	78	67	111		0.18	0.72	0.6	31	124	26	291
0.4	0.12	2.3	15	64	55	114		0.06	0.00					
0.5	2.92	1.0	10	68	46	116		0.11	0.67					
0.4	0.12	4.1	24	61	62	123		0.13	0.02					
0.4	0.04	1.2	23	77	42	175		0.11	0.01					
0.5	0.20	1.5	42	105	69	278		0.13	0.15					
